# A Tapered Aluminium Microelectrode Array for Improvement of Dielectrophoresis-Based Particle Manipulation

**DOI:** 10.3390/s150510973

**Published:** 2015-05-11

**Authors:** Muhamad Ramdzan Buyong, Farhad Larki, Mohd Syafiq Faiz, Azrul Azlan Hamzah, Jumrail Yunas, Burhanuddin Yeop Majlis

**Affiliations:** Institute of Microengineering and Nanoelectronics (IMEN), Universiti Kebangsaan Malaysia, Bangi, Selangor 43600, Malaysia; E-Mails: farhad@ukm.edu.my (F.L.); muhammadelfaiz@gmail.com (M.S.F.); azlanhamzah@ukm.edu.my (A.A.H.); jumrilyunas@ukm.edu.my (J.Y.); burhan@vlsi.eng.ukm.my (B.Y.M.)

**Keywords:** dielectrophoresis, taper microelectrode, CMOS, numerical simulation, array

## Abstract

In this work, the dielectrophoretic force (F_DEP_) response of Aluminium Microelectrode Arrays with tapered profile is investigated through experimental measurements and numerical simulations. A standard CMOS processing technique with a step for the formation of a tapered profile resist is implemented in the fabrication of Tapered Aluminium Microelectrode Arrays (TAMA). The F_DEP_ is investigated through analysis of the Clausius-Mossotti factor (CMF) and cross-over frequency (f_xo_). The performance of TAMA with various side wall angles is compared to that of microelectrodes with a straight cut sidewall profile over a wide range of frequencies through FEM numerical simulations. Additionally, electric field measurement (EFM) is performed through scanning probe microscopy (SPM) in order to obtain the region of force focus in both platforms. Results showed that the tapered profile microelectrodes with angles between 60° and 70° produce the highest electric field gradient on the particles. Also, the region of the strongest electric field in TAMA is located at the bottom and top edge of microelectrode while the strongest electric field in microelectrodes with straight cut profile is found at the top corner of the microelectrode. The latter property of microelectrodes improves the probability of capturing/repelling the particles at the microelectrode’s side wall.

## 1. Introduction

Dielectrophoresis (DEP) is a method for particle replacement as a result of its dielectric properties. The initial work on this phenomenon for implementing it as a separation tool for suspended particles in an organic medium was done by Pohl [1,2]. In this technique a sinusoidal time varying and spatially non-uniform electric field is implemented to manipulate the position of a particle as a result of its dielectric properties. DEP enables focusing, translation, and trapping as well as the characterization, purification, and enrichment of a wide range of materials such as environmental, biological and clinical analytes within a fluid suspending medium [3,4,5]. The developing of contactless, marker and label free manipulation research via integration of dielectrophoretic microelectrodes into a lab-on-a-chip reveals further the potential applications of dielectrophoresis in nano/micro-machines [6,7,8,9]. Indirect physical contact or contactless particle movement as found many applications in areas such as drug discovery and delivery applications as well as disease screening, and separation and biological sample analysis [10,11,12,13]. This is mainly due to the ability of indirect physical contact movement to eliminate any consequent contact damage and related problems compared to direct physical contact. In fact, it becomes more challenging when the subject of movement is of a few microns or nano sized, which is unworkable to handle with direct physical contact. Thus, the advantage of this method for contactless movement of particles can eliminate the impact of the initiation of physical contact. For these reasons, the movement via indirect physical contact using dielectrophoretic properties is proposed. Other techniques such as fluorescence activated cell sorting (FACS) [14,15], magnetic activated cell sorting (MACS) [16,17], and field flow fractionation (FFF) [18,19,20] can be also used for the movement and separation of particles and particularly cells, but particle and cell separations based on DEP force (F_DEP_) using dielectric polarization have better reliability and capability performance in terms of sensitivity and selectivity. Additionally, in terms of setup, DEP uses the simplest setup compared to magnetic, mechanical, hydrodynamic, optical and field flow fractionation methods [21]. Based on the configuration of microelectrodes, Khoshmanesh *et* *al.* [5] classified DEP devices as follows: parallel or interdigitated [22,23], castellated [24,25], oblique [26], curved [27,28], quadrupole [29,30], microwell [31,32], matrix [33], extruded [34], top-bottom patterned [35,36], insulator-based or electrodeless [37], and contactless [38,39].

In this work, a new microelectrode profile is introduced to enhance the sensitivity and selectivity of the F_DEP_ technique by introducing a more non-uniform electric field in the medium. The device is designed based on microelectrode arrays with a tapered profile which we named as Tapered Aluminium microelectrode arrays (TAMA), fabricated using the standard CMOS processing technique. Standard CMOS processing technique is a mature technology regarding cost effectiveness, reliability and manufacturability as well as integration capability [40]. The F_DEP_ on particles was further analyzed based on its force strength and direction through experimental measurements and COMSOL Multiphysics numerical simulation of device. First, the F_DEP_ is investigated based on the Clausius-Mossotti factor (CMF) and cross-over frequency (f_xo_) from direct experimental measurements. Then, the Finite Element Method (FEM) is implemented to compare the field profile in tapered electrodes with different microelectrode angles varying from 10° to 90° (straight cut profile). Finally, the field profile in tapered and straight cut microelectrodes is compared through the electric field measurement (EFM) technique by atomic force microscopy (AFM). The proposed device can be used as the fast and easy tool for cell/particle manipulation as well as for investigating the electrical properties of particles and living cells in a given environment.

## 2. Theoretical Background of F_DEP_

The time-averaged DEP force (F_DEP_) applied on a spherical particle is obtained as below [41,42]:

(1) <F> =2πεoεmR3Re[CMF]∇E2+4πr3ε0εmIm[CMF]∑x,y,zE2∇ϕ

where ε_o_ is the permittivity for vacuum 8.854 × 10^−12^ F/m, ε_m_ is the relative permittivity of the suspending medium, R is the radius of the particle, CMF is Clausius–Mossotti factor, E is the root-mean-square value of the applied electric field, and φ is the phase component of the electric field. 

The first term is called ‘classical DEP force’ (F_DEP_) and is proportional to the real part of the CMF (in-phase component of the electrical polarization induced in the particle) and is related to the spatial non-uniformity of the electric field. According to the positivity or negativity of Re (CMF) value which reflects whether the particles polarize more or less than their suspending medium in the applied field, F_DEP_ causes particles to move toward strong or weak field regions. Alternatively, the second term which is called travelling wave (TW) DEP force (F_TW_-DEP) is directly proportional to the imaginary part of the CMF which is out-of-phase component of the particle polarization. F_TW_-DEP is related to the speed which the electric field distribution is traveling and reflected by the electric field phase gradients. The DEP response of the particle depends on the resultant current that lies in-phase with the applied field, and this is proportional to the real component (Re [CMF]) value of the polarizability parameter CMF in Equation (1) [21]. 

The CMF, which describes the relative polarization of a particle with respect to the surrounding medium, is a geometry and frequency dependent variable that for spherical particles is given by:

(2) $$\text{CMF (}\varepsilon_{\text{particle}}\text{, }\varepsilon_{\text{medium}}\text{, }\sigma_{particle}\text{, }\sigma_{medium}\text{, }\omega \text{) }=\frac{\left(\varepsilon_{\text{particle}}-\varepsilon_{\text{medium}})+i/\omega (\sigma_{\text{particle }}\text{ -}\sigma_{\text{medium}}\right)}{\left(\varepsilon_{\text{particle}}+2\varepsilon_{\text{medium}})+i/\omega (\sigma_{\text{particle }}\;+\sigma_{\text{medium}}\right)}$$

According to Equation (2) two limiting cases can be revealed as:

(A) For low frequency applications:

(3) $$\underset{\omega \to \text{0}}{\text{lim}}\text{[CMF] }=\frac{\left(\sigma_{\text{particle }}\text{ -}\sigma_{\text{medium}}\right)}{\text{ (}\sigma_{\text{particle }}\;+\text{ 2 }\sigma_{\text{medium }}\text{)}}$$

where σ_particle_ and σ_medium_ are the conductivities of the particle and suspending medium, and:

(B) For high frequency applications:

(4) $${\varepsilon^{*}}_{\text{medium }}\;=\varepsilon_{0}\varepsilon_{\text{medium }}-\frac{i\sigma_{\text{medium}}}{\;\omega },\;{\varepsilon^{*}}_{\text{particle }}\;=\varepsilon_{0}\varepsilon_{\text{particle }}-\frac{i\sigma_{\text{particle }}}{\;\omega }$$

where ε_particle_ and ε_medium_ are the absolute permittivity of the particle and suspending medium, respectively and $i=\sqrt{-1}$. 

It can be seen that the sign of the CMF can be determined by the electrical conductivities of the particle and the medium at low frequencies. However, it is determined by the permittivity at higher frequencies.

## 3. Experiments and Methods

### 3.1. Microelectrode Fabrication 

**Figure 1:**
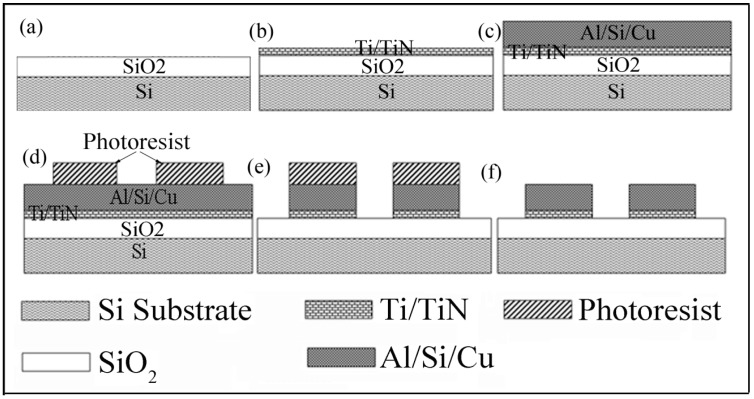
Schematic of the process flow for TAMA fabrication.

The CMOS processing technique is implemented in the fabrication process of the TAMA platform on a silicon substrate. The fabrication of the TAMA platform is started with deposition of 1.15 µm silicon oxide (SiO_2_) as an insulator layer on top of a silicon substrate by means of plasma-enhanced-chemical-vapor-deposition (PECVD). A thin adhesion layer of titanium/titanium nitrite (Ti/TiN) with thickness of 60 nm/30 nm is deposited using physical-vapour-deposition (PVD). Following the Ti/TiN deposition a layer of aluminium/silicon/copper Al/Si/Cu (98/1/1 wt%) with thickness of 4.0 µm is deposited using PVD. Photolithography with resist thickness of 4.0 μm including a UV curing for hardness photoresist process is executed to transfer the square array design onto the Al/Si/Cu layer. In the final step, Al/Si/Cu is etched using an inductive coupled plasma (ICP) etcher for metal etching with an advance plasma resist strip. A schematic of the fabrication steps are presented in Figure 1a–f.

**Figure 2:**
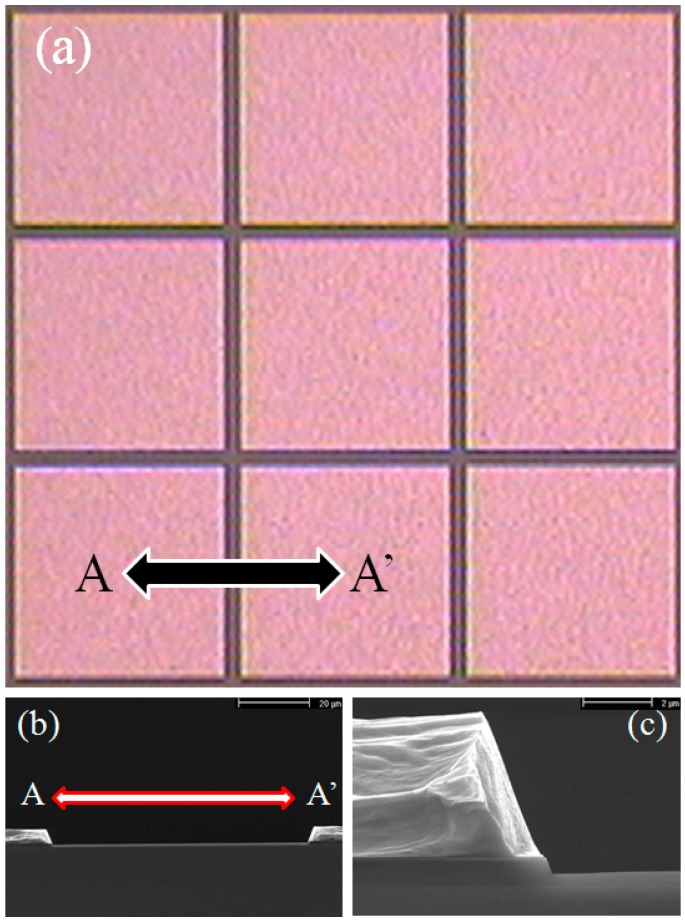
(**a**) Top view image of TAMA; (**b**) SEM image of a cross sectional view; and (**c**) blown up image of the microelectrode.

Prior to the final Al/Si/Cu etch step, and after the lithography process an additional resist taper profile formation step was implemented. The new combination of the resist profile process and etching technology are found to give desired taper microelectrode profile angle. Using a combination of resist plasma etching by reactive ion etching (RIE) and metal etching via ICP methods, the desired tapered microelectrode profile has been produced. The top view of the square array microelectrodes and a scanning electron microscope (SEM) image of a cross sectional view of the two electrodes and a blow up of a single electrode for TAMA are presented in Figure 2a–c, respectively. The space gap on each side is 80 µm and square array microelectrodes are 1100 × 1100 µm. The tapered profile on the side wall of the TAMA microelectrode can be clearly observed from the SEM images shown in Figure 2b,c. It should be noted that, this profile is intentionally formed to produce the highest electric field gradient with the most selectivity at the bottom of the sidewall microelectrode and to help particles lean toward the sidewall and be trapped.

### 3.2. Measurement Equipment and Methods

#### 3.2.1. Particle Preparation

**Figure 3:**
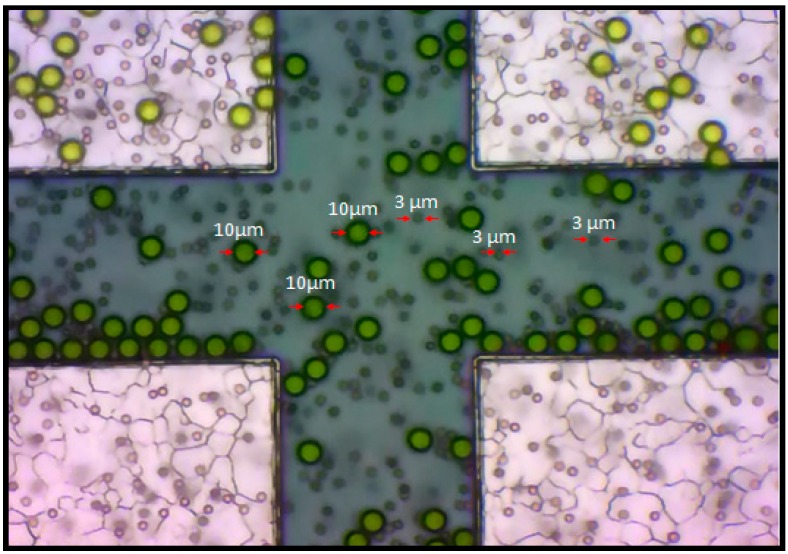
Fluor-max aqueous fluorescent particles with 10 and 3 µm diameter.

The analysis of the strength and direction of the F_DEP_ was conducted using Thermo Scientific TM Fluor-max aqueous fluorescent particles (Fluoro-Max Dyed, Thermo Fisher Scientific Inc., Waltham, MA, USA).These particles emit bright and distinct colors (green and red) when illuminated by light of an appropriate wavelength, which improves their contrast and visibility relative to other materials in the background. Consequently, they can be used to efficiently improve the sensitivity and detectability during our analysis. Fluor-max aqueous fluorescent particles, referred to as engineered particles in further discussion, are internally dyed polystyrene microsphere suspensions which are fluorescent green for 10 µm diameter and fluorescent red for 3 µm diameter, as shown in Figure 3. 

#### 3.2.2. Experimental Setup

**Figure 4:**
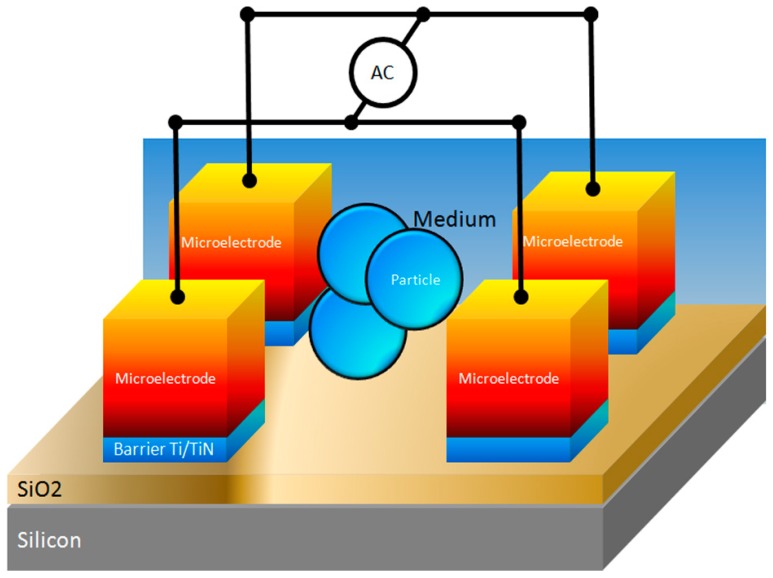
Schematic view of the experimental setup.

A schematic view of the experimental setup is shown in Figure 4. The characterization of the experimental work for the TAMA is performed using a micromanipulator with a standard prober system (Micromanipulator Co, Inc., Carson City, NV, USA). Sinusoidal electrical signals from a function generator (IWATSU SG-4105 (10 V peak to peak, 15 MHz) are directly connected to the prober to supply voltage of various frequencies to the microelectrode pad. 

This micromanipulator is designated for electrical testing purposes and is equipped with a standard microscope without a video camera. Therefore, an additional eye-piece microscope camera (Dino-Eye, Hsinchu, Taiwan) is attached to the eye-piece microscope of the micromanipulator for video recording. This configuration setup is suitable for opaque substrates such as the silicon substrate used for the TAMA. In addition, for more accurate measurement of particle velocities a high speed camera (Phantom V 7.3, AMETEK, Wayne, NJ, USA) integrated with confocal scanner unit (CSU 22 Yokogawa, Tokyo, Japan) microscope that uses a laser as a source light (Melles Griot Argon Ion Laser System IMA 100). At 800 × 600 resolution, the Phantom V 7.3 shoots up to 6688 frames-per-second. The Phantom V 7.3 offers global on-chip shuttering to 1 µs (fixed at 1 µs in Turbo Mode). Using the PIVTEC software we were able to perform imaging processing analysis for determine the particle velocity. 

**Figure 5:**
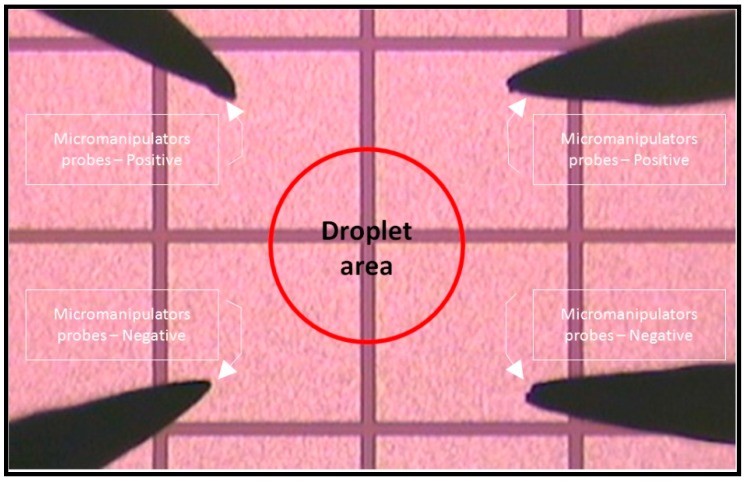
Setup for visualizing and recording the F_DEP_. The probes’ location and the droplet area are indicated.

Figure 5 shows the setup for visualizing and recording the F_DEP_ by utilization of the micromanipulator stages and probes. The regions corresponding to four microelectrodes and droplet areas are indicated. Four quadrant microelectrodes are separated into four zones, the top left and right corner of the microelectrode are connected to the positive polarity of the source node while the bottom left and right corner of the microelectrode are connected to the ground node. Utilization of a precision syringe (80401 25 µL syringe, Hamilton, Reno, NV, USA) to produce a 10 µL droplet of an evenly mixed mixture of engineered particles with 10 and 3 µm sizes are dispensed on the top of the microelectrode surface to visualize the F_DEP_ behavior. Thirty tests were run using a similar input voltage of 5 V peak-to-peak with a frequency in the range of 1 Hz to 1.0 GHz, in ×10 Hz increment steps was applied to the microelectrode for a period of up to 120 s per test run.

#### 3.2.3. Determination of CMF

In order to quantify the F_DEP_, several direct and indirect methods are available such as particle counters, collection rate measurements, measurements of the levitation height, and particle velocity measurements [43]. In this work, our focus was on particle velocity measurements. According to Equations (3) and (4), indirect methods based on the conductivity and permittivity of particle and medium two cases can be considered: (i) if $\sigma_{\text{particle}}<\sigma_{\text{medium}}$ and $\varepsilon_{\text{particle}}<\varepsilon_{\text{medium}}$, the CMF is positive (*i.e*., P_DEP_) at high frequencies and negative (*i.e*., N_DEP_) at lower frequencies, and this behavior is reversed for the case of (ii) $\sigma_{\text{particle}}>\sigma_{\text{medium}}$ and $\varepsilon_{\text{particle}}$ > $\varepsilon_{\text{medium}}$. It should be noted that, since CMF is a function of the complex permittivity of the particle and the media its value partially determines the magnitude of the force and its direction. 

In direct method of CMF determination an imaging analysis of the velocity measurement of the particle (*U_part_*) in a fluid with viscosity of η is implemented by assuming that the particle motion is quasi-static and DEP force is balanced by Stokes’s drag under low Reynolds number conditions. The obtained velocity value is then inserted into the following formula to calculate the Re (CMF):

(5) Re[CMF]=αUpart where α=3ηR2εm∇|E|2

**Figure 6:**
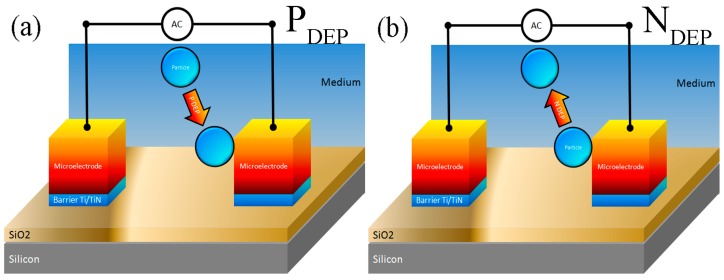
Schematic illustration for (**a**) positive DEP (P_DEP_) and (**b**) negative DEP (N_DEP_).

In out experimental work analysis on the CMF, we followed the techniques reported in [44,45] which implement two steps for CMF measurement. In the first step we obtained CMF at P_DEP_, which is when the particle at the centre of the microelectrode array moves towards to the edge of the microelectrodes where the region high electric field is. Particle movement is directed from the lower electric field zone to the highest electric field one since the particles are more polarized than the medium (Figure 6a). In the second step we obtained CMF at N_DEP_, which is done by movement of particles concentrated in the center of the microelectrode toward the edges of the microelectrode (P_DEP_) and then by applying an appropriate frequency so these particles moved far away from the edge of the microelectrode towards to the centre of the subsequent microelectrode. In this case, particle movement is directed from the higher electric field zone to the lower electric field one as the medium is more polarized than the particle (Figure 6b).

If the electrical conductivity of the particle is equal to that of the medium the real part of CMF has a value equal to zero. During the transition, the DEP response switches between N_DEP_ and P_DEP_. The point where the N_DEP_ response switches to the P_DEP_ one (or the P_DEP_ response switches to N_DEP_) is called the cross-over frequency (f_xo_). A direct method of determining f_xo_ via an experimental measurement technique is obtained by monitoring the P_DEP_ and N_DEP_ of particle movement responses. To validate this technique, we monitored two transitions from N_DEP_ to P_DEP_ and from P_DEP_ to N_DEP_. When subjected to the input applied frequency, f_xo_ is found in the transition between N_DEP_ to P_DEP_ or P_DEP_ to N_DEP_.

#### 3.2.4. Numerical Simulation

**Figure 7:**
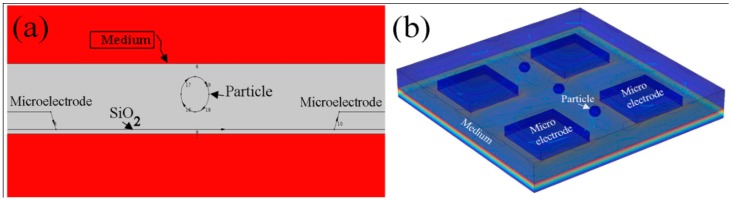
Schematic views of COMSOL FEM Model, (**a**) 2D; (**b**) 3D.

To scrutinize the magnitude of the F_DEP_ in microelectrodes with tapered and straight cut profiles, we have performed a finite element method (FEM) simulation using the COMSOL Multiphysics software package (ver. 4.2a, Los Angeles, CA, USA). Due to the constraints of the microelectrodes’ actual size and the computational time for the simulations of the electric field between two microelectrodes, our microelectrode actual size simulation is simplified and deduced with the support of data from the whole spectrum. All simulations were conducted as two dimensional (2D) approximations. In order to examine the accuracy of the 2D simulation, we compared results with three dimensional (3D) simulations in term of the spatial distribution of the electric field on the particle. Both the 2D and 3D simulation results agree in the AC/DC module using the electrostatic model. The geometry and boundary conditions of the microelectrode profile and particle used in the 2D and 3D FEM model are illustrated in Figure 7a,b.

The array type of dielectrophoretic microelectrodes is modeled in a square geometry. Aluminium (Al) is considered as the microelectrode material and the angle is varied from 10° to 90°. The length and width is 10 µm with a thickness of 2 µm and the distances between the electrodes are assumed as 80 µm. A layer of SiO_2_ with thickness of 1 µm which works as an insulator layer between microelectrode array and Si substrate is considered. The spherical particles are modeled using a borosilicate material. A continuous-phase liquid (water) containing dispersed particles with a height of 15 µm covered the entire microelectrodes and insulator layer. For all selected materials the standard electrical and physical properties such as the relative permittivity, electrical conductivity and water viscosity as provide by software’s library were used. A quasi-static potential field was simulated for surface potentials of electrodes with 5 V applied to the source electrode while the other electrode is grounded. In this work, the 2D and 3D simulations share equivalent parameters.

## 4. Results and Discussion

**Figure 8:**
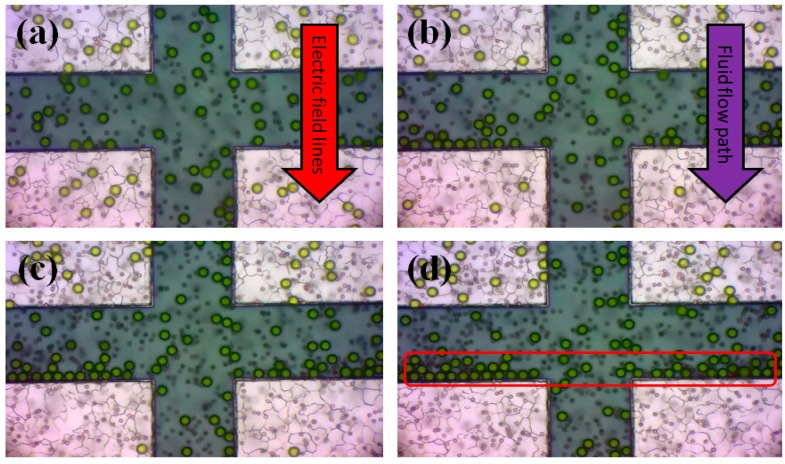
Trapping of Fluoro-max dyed particles of 10 µm diameter (green particles) by TAMA after (**a**) 30 s; (**b**) 60 s; (**c**) 90 s; and (**d**) 120 s.

Investigation of the particles movement by F_DEP_ is mainly based on the attraction towards the higher gradient region (P_DEP_) or the repulsion towards the lower gradient region (N_DEP_). There are two main parameters that must be considered. First, it is necessary to obtain an appropriate approximation for the applied input frequency for attraction and repulsion of particles in the medium. This indicates the relationship between polarisable particles and medium which is defined as CMF. The second step is obtaining the cut-off frequency (f_xo_) at the intercept of CMF values which is the value of the transition frequency from N_DEP_ to P_DEP_ and P_DEP_ to N_DEP_. While monitoring the migration of the particles, the geometry profile of the microelectrode sidewall is capable of improving the sensitivity and selectivity. This statement is supported by the direct CMF method experimental work results. Trapping of Fluoro-max dyed particles of 10 µm diameter (green particles) after 30, 60, 90 and 120 s is shown in Figure 8a–d. Particles trapped at edge of the TAMA are highlighted in Figure 8d. 

**Figure 9:**
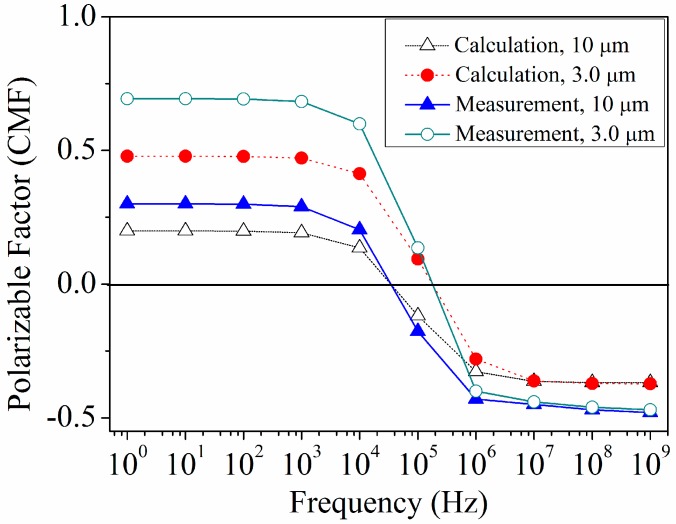
Measured and calculated frequency dependence of the real part of the CMF for 10 and 3 µm engineered particles.

It should be noted that the Fluoro-max dyed particles of 3 µm diameters (red particle) remain un-trapped at this particular frequency.We experimentally observed that the 3 µm particles are dispersed all over, while 10 µm particles were collected at the microelectrode edges as highlighted in Figure 8d. Figure 9 presents the measurement results based on Equation (5) and the calculated results according to Equation (3) for the frequency dependence of the real part of the CMF for 10 and 3 µm engineered particles. In the calculation, the fluid medium (DI water) had a conductivity and relative permittivity of 0.0002 S/m and 78, respectively. The relative permittivity of the engineered particles was 2.5 and the overall conductivities of the 3 and 10 μm engineered particles were equal to 7.5 × 10^−4^ S/m and 3.5 × 10^−4^ S/m, respectively. The 10 and 3 µm particles experience a positive DEP force when the frequency of the applied AC field is below 0.02 MHz and 0.1 MHz, respectively while a negative DEP force acts on the 10 and 3 µm particles when the frequency is above 0.02 and 0.1 MHz. The overall comparison between the calculated and measurement results of CMF and f_xo_ for 10 and 3 µm engineered particles with the TAMA microelectrode platform indicates a similar trend. However, it can be observed that the F_DEP_ strength obtained via measurements is higher than the calculated value. We observed a deviation leading to the overestimation of the particle velocities due to the measurement error which is defined as the limitation of the velocity measurements through the optical microscope.

In order to compare the strength, direction and distribution of the electric field in the microelectrodes and obtain the optimized taper angle for the most effective influence on the particles in the medium, a FEM numerical simulation for microelectrodes with angles varying from 10° to 90° is performed in increments of 10°. 

**Figure 10:**
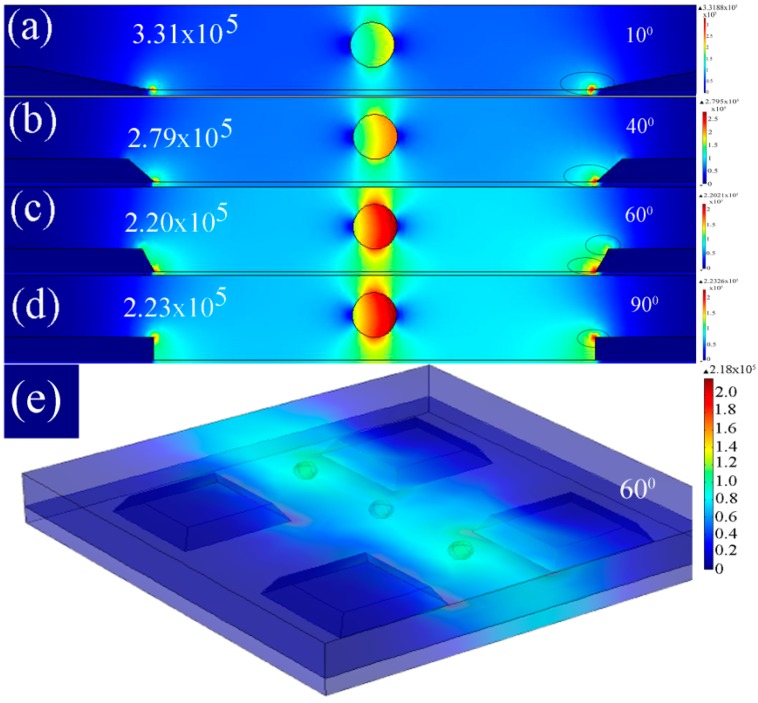
2D FEM analysis of the electric field gradients for microelectrodes with a distance of 80 µm, and (**a**) 10° (**b**) 40° (**c**) 60° (**d**) 90° side wall angle; (**e**) 3D FEM analysis of the electric field gradients for microelectrodes with 60° side wall angle.

Figure 10a–d shows the effect of angle variation on the electric field distribution and consequently its impact on the particles in the medium. The area of the highest electric field is also indicated for microelectrodes with 10°, 40°, 60° and 90° taper profile. Although the structure with 10° taper profile produce the strongest electric field, the impact on the particle was not as significant as that of the structure with taper angles between 60° and 90°.

**Figure 11:**
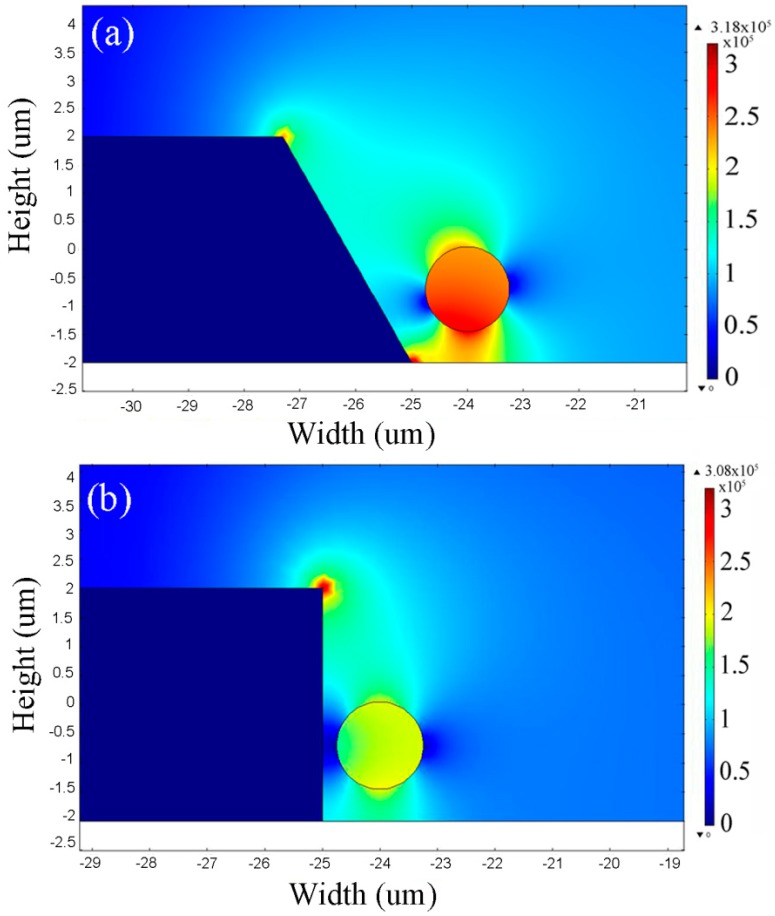
2D FEM analysis of the electric field gradients for microelectrodes with (**a**) 60° (**b**) 90° side wall angles. The particle is located near the microelectrode edge.

Furthermore, compared to the microelectrode with 90° profile (straight cut) the structures with 60° and 70° profiles produce the highest electric field in two different regions (top edge and bottom edge) which produces a more effective non-uniform electric field in the medium. It should be highlighted that in the structure with a straight cut profile the electric field takes the path of least resistance at the top of the microelectrode edge, while it takes the path of least resistance at the top and bottom edges of the microelectrode in TAMA. In Figure 10e, a 3D simulation of microelectrodes is shown for comparison with the 2D simulation results. It can be seen that the electric field profile presents the same trend with the 2D simulation. The effect of the microelectrode sidewall on particles is more significant when the particle is near the sidewall edge. In Figure 11a,b microelectrodes with two different side wall profiles (60° and 90°) are presented when the particle is located near the sidewall. The stronger effect of the tapered profile compared to the straight cut structure can be observed clearly by considering the electric field distribution around the particle. 

**Figure 12:**
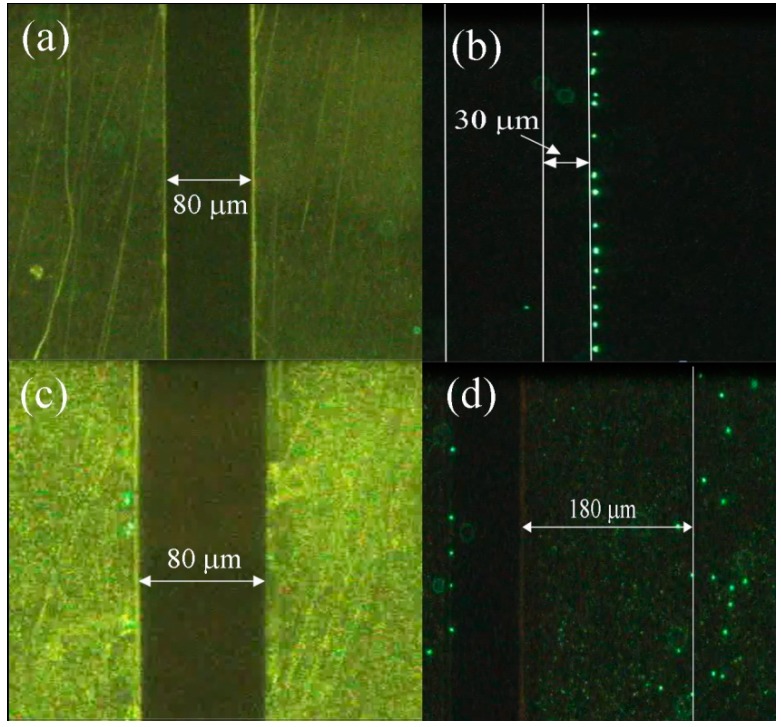
Top-view observation (X-Y plane) of the particle displacement for two different microelectrode profiles: (**a**) microelectrode with 90° sidewall profile before introducing particles; (**b**) microelectrode with 90° sidewall profile after introducing particles; (**c**) microelectrode with 70° sidewall profile before introducing particles; and (**d**) microelectrode with 70° sidewall profile after introducing particles. The applied voltage is 5 V and the applied frequency is 1 MHz.

In order to confirm the effect of the microelectrodes’ sidewall profile, two different structures with 70° and 90° profile are investigated experimentally. Figure 12a,b presents top-views of the microelectrode with 90° profile before and after introducing particles, respectively. After introducing particles to the medium a 5 V voltage at 1 MHz frequency is applied to the microelectrodes. The microelectrodes’ border and the maximum particle displacements are highlighted in lines on Figure 12b. It can be seen that due to the N_DEP_ force, the particles can be repelled to approximately 30 μm from the microelectrode’s edge (the distance between electrodes is 80 μm). Applying the same voltage and frequency to the microelectrode with 70° angle (Figure 12d reveals that the particles can go as far as 180 μm from the microelectrode edge which is a direct consequence of the stronger dielectrophoretic force created by the microelectrode.

**Figure 13:**
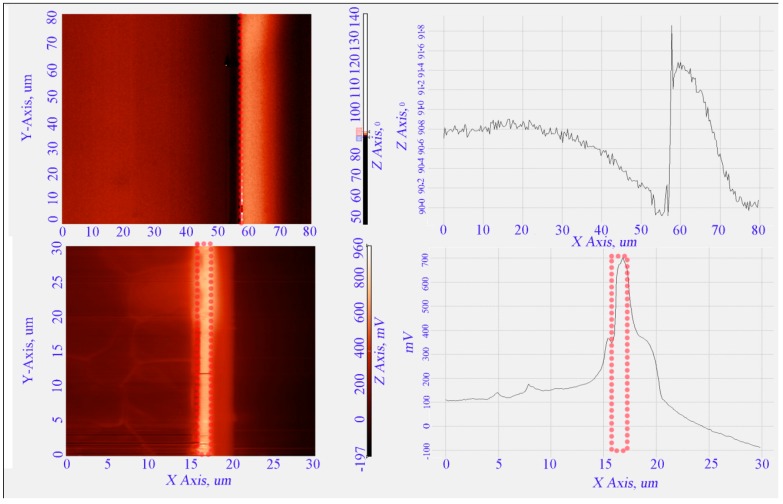
Electric field profile of straight cut and tapered microelectrodes: (**a**) top view of straight cut and (**b**) line profile of straight cut microelectrodes (**c**) top view of tapered and (**d**) line profile of tapered microelectrodes.

Further analysis of the electric fields is implemented through the electric field measurement (EFM) technique using atomic force microscopy (AFM). A Scanning Probe Microscope (NT-MDT NTEGRA Prima, Moscow, Russia) is used to indicate the highest spot of the electric field for straight cut and tapered microelectrodes. The measurements using the electric field measurement (EFM) technique via many pass scanning and image analysis using the P9 software for the two structures are shown in Figure 13a,d. The Image Analysis P9 program serves for processing and analyzing SPM images and data. The program provides a wide variety of techniques to process and analyze both SPM images and related 2D and 1D functions of data such as analysis of the surface profile in a desired section, analysis of the surface roughness, spectral analysis, and spatial filtration of images with a number of predefined filters.In microelectrodes with a straight cut profile the analysis (Figure 13a) top view image gives insufficient information, however the line profile (Figure 13b) indicates that the highest electric field most probably appears on the top edge of the microelectrode. On the other hand, in the TAMA structure analysis of the electrical field from the top to the bottom of the tapered profile in top view (Figure 13c) and line profile (Figure 13d) indicate that the electric field gradually increases from the top to the bottom corner of the microelectrode and the spot with the strongest electric field appears at the bottom. The electrical field trends for straight cut and tapered microelectrodes are in agreement with the FEM analysis. 

By considering the role of input frequency to increase the sensitivity and selectivity and the microelectrode profile impact to increase the F_DEP_ on particles one can obtain the optimized parameters for further analysis. By careful investigation of the FEM and experimental results of the TAMA structures in terms of electrical field strength and direction, at optimal condition of applied frequency and microelectrode profile, we have pointed out that the thickness of tapered microelectrodes might also play a critical role and has a correlation with the diameter of particles with the intention to increase the trapping efficiency rate. 

## 5. Conclusions

We have demonstrated TAMA as a new platform for improving the sensitivity and selectivity of dielectrophoretic force (F_DEP_). The TAMA consists of an Al microelectrode array on a Si substrate and are fabricated based on the CMOS processing technique. F_DEP_ in TAMA is investigated with respect to the variation of CMF and f_xo_ in a wide range of frequencies. The analysis of experimental measurements, FEM simulation and EFM technique by AFM of TAMA with different sidewall profile angles indicated higher trapping rate efficiency in TAMA with sidewall profiles between 60° and 70°. According to the electrical field analysis it is also concluded that, compared to the straight cut profile which produces the electrical field at the top of the microelectrode edge, the tapered microelectrode profile produces a higher gradient non-uniform electric field from the top and bottom edges of the microelectrodes. We believe that the TAMA concept can be further explored to investigate the effect of non-uniform electrical fields that are related to the magnitude and direction of F_DEP_ on the functional activity of sensitive and selectivity polarization as a mechanism to transport, accumulate, separate and characterize micro/nano scale particles. Therefore, it could enable the inexpensive, fast, highly sensitive, highly selective and label-free detection and analysis of target particles.
